# Analytical validation of a novel bioassay for thyroid-stimulating immunoglobulin

**DOI:** 10.3389/fendo.2024.1468768

**Published:** 2025-01-07

**Authors:** Paul D. Olivo, Hannah Kim, Lynn Miao, Jeffery A. Houtz, George J. Kahaly

**Affiliations:** ^1^ Department of Microbiology and Microbial Pathogenesis, Washington University Medical School, St. Louis, MO, United States; ^2^ QuidelOrtho Corporation, San Diego, CA, United States; ^3^ Molecular Thyroid Research Laboratory, Department of Medicine I, Johannes Gutenberg University (JGU) Medical Center, Mainz, Germany

**Keywords:** analytical performance, validation, bioassay, thyroid-stimulating receptor antibody, thyroid-stimulating immunoglobulin

## Abstract

**Background:**

A novel and rapid cell-based bioassay, Turbo TSI, for measurement of thyroid-stimulating immunoglobulins (TSI) was recently reported. An assessment of the analytical performance of this TSI bioassay is described herein.

**Methods:**

Thawed cells from Turbo TSI kits were treated with different concentrations of a World Health Organization (WHO) international standard (IS) TSI-positive serum. TSI was measured as a function of luciferase activity measured as relative light units (RLU) and converted into international units per liter (IU/L). Analytical performance studies were performed on numerous samples, over multiple days, by two users at two sites.

**Results:**

The limit of blank, limit of detection and limit of quantitation were determined to be 0.007 IU/L, 0.014 IU/L, and 0.021 IU/L, respectively. Receiver operator characteristics (ROC) analysis determined the cut-off to be 0.0241 IU/L with an area under the curve of 0.984. The linear range was shown to be from 0.015 to 11.958 IU/L. The intra-laboratory precision was ≤15%CV. The overall reproducibility of the assay was ≤20%CV for five concentrations (0.06 to 5.16 IU/L). Interference and cross reactivity studies with a variety of substances showed that the assay was robust. The Turbo TSI bioassay demonstrated 95.2% (95% CI 83.3-98.1) positive percent agreement and 94.8% (95% CI 90.9-97.1) negative percent agreement with an FDA-cleared bioassay (Thyretain**^®^** TSI) using serum from 295 patients with autoimmune thyroid disease.

**Conclusions:**

The Turbo TSI bioassay exhibits excellent analytical performance and a high level of reproducibility. The performance compared well with Thyretain**^®^** TSI, an FDA-cleared TSI bioassay.

## Introduction

Both guidelines of the European (ETA) and American (ATA) thyroid associations for the management of thyrotoxicosis in general and autoimmune hyperthyroidism in particular, as well as the guidelines for the management of Graves’ eye disease, emphasize the role of the thyrotropin receptor autoantibodies (TSH-R-Ab) in the management of patients with subclinical and overt hyperthyroidism (1–3). These guidelines also recommend the measurement of these disease-specific autoantibodies for the accurate diagnosis and monitoring of the clinical activity and severity of the autoimmune thyroid and extrathyroidal diseases. Indeed, the role of stimulatory anti-TSH-R-Ab in the pathogenesis of autoimmune hyperthyroidism is well established (4–6). Only bioassays can differentiate the functionality of and measure the stimulatory activity of anti-TSH-R-Ab (7, 8). Many bioassays for TSI have been described including an FDA-cleared TSI bioassay (Thyretain**^®^**, QuidelOrtho, San Diego, CA) (9). Validation and standardization of this established and reproducible bioassay have been reported (10, 11). Furthermore, dilution analysis of TSI positive samples yielded predictive values pertaining to response to antithyroid treatment and proved to be able to differentiate between various Graves’ phenotypes (12–14).

Recently, a novel bioassay was developed which greatly reduces the complexity and time required to measure TSI. This new bioassay, referred to as Turbo TSI, avoids the need for cell culture, has no wash or lysis steps, and has a total turn-around-time of less than 2 hours (15). Preliminary performance data show that it performed well in detecting and measuring levels of TSI. The present study aimed to evaluate the analytical performance of the Turbo TSI bioassay for the detection of TSI.

## Materials and equipment

### Cells

The GS-TSH-R Mc4 cell line used in the Turbo TSI bioassay in the present study were obtained from QuidelOrtho Corporation, San Diego, CA. The GS-TSH-R Mc4 cells are Chinese hamster ovary (CHO) cells genetically engineered to express a chimeric form of the human TSH-R and a modified luciferase enzyme, Glosensor (GS), that is active only in the presence of cyclic AMP (15). Frozen cells are thawed immediately prior to use in the bioassay.

### Turbo TSI stimulating reporter bioassay kit reagents

- Turbo TSI Cells.
- Turbo TSI Negative Control (Neutral Cap).
- Turbo TSI Low Positive Control (White Cap).
- Turbo TSI Mid Positive Control (Brown Cap).
- Turbo TSI High Positive Control (Black Cap).
- Turbo TSI cAMP Reagent.
- Turbo TSI Standard A (Red Cap).
- Turbo TSI Standard B (Orange Cap).
- Turbo TSI Standard C (Yellow Cap).
- Turbo TSI Standard D (Blue Cap).
- Turbo TSI Standard E (Purple Cap).
- Turbo TSI Data Analysis Software.

### Other materials and equipment required

–70°C or lower freezer or liquid nitrogen Dewar.
- Luminometer capable of reading a 96 multi-well plate (e.g. GloMax Navigator, Promega, Madison WI)).
- Luminometer Calibrator Plate.
- Sterile Transfer Pipette.
- 96-Well White, Flat Bottom Assay Plate (e.g., Costar, 3912).
- Microplate Adhesive Film (e.g., USA Scientific, 2920-0000).
- Sterile Reagent Reservoirs (e.g., VWR, 41428-954).
- Water Bath, 35°C to 37°C.
- Vortex Mixer.
- Timer.
- Household Bleach.

## Methods

### Bioassay protocols

The Thyretain**^®^** TSI bioassay was performed according to the manufacturer’s product insert and as previously described (14, 16).

### Brief overview of Turbo TSI protocol/procedure

Briefly, TSI standards, controls, and test samples are added to a white 96-well white plate in 5 µL per well. A freezer vial containing Turbo TSI cells at a concentration of 4x10^6^ cells/mL is thawed in a 37°C water bath. These cells are then mixed with 5 mL of cAMP reagent with luciferase substrate (Promega, Madison, WI, USA) and placed in a reagent reservoir. The cell suspension is then dispensed into the wells of the plate at 50 µL/well. The plate is then incubated at ambient temperature for 60 minutes. At the end of the incubation, the plate is read using the GloMax Navigator luminometer (Promega, Madison, WI). The resulting relative light units (RLU) are then converted to TSI quantity in IU/L using the Thyretain**^®^**/Turbo TSI Data Analysis Tool.

### Detailed steps of Turbo TSI procedure

1. Thaw one vial of each cAMP Reagent, Standard Panel, and Controls for 7 to 10 minutes in a 35°C to 37°C water bath. Ensure these reagents are equilibrated to room temperature (20°C to 25°C).
2. Vortex samples, standards, and controls for 5 to 10 seconds. Add 5-μL Standards, Control, or sample to the bottom corner of each well in a white 96-well plate in singlet.
3. Thaw one vial of Turbo TSI Cells for 3 to 5 minutes in a 35°C to 37°C water bath until just thawed.
4. Transfer the entire volume of the cells with a transfer pipette to the bottle containing 5-mL of cAMP Reagent. *(Note: The cell suspension must be added to the cAMP Reagent within 30 minutes of thawing).*
5. Mix by inverting the bottle several times.
6. Dispense 50-μL per well of the Turbo TSI cell suspension (Step 4) per well using a 20-μL to 200-μL (or 50-μL to 300-μL) multi-channel pipette. (*Note: The cell suspension must be added to the plate within 30 minutes after combining with the cAMP Reagent)*.
7. Seal the plate with a microplate adhesive film. Gently shake the plate laterally right to left on the benchtop 5 to 6 times.
8. Incubate the plate at room temperature for 60 minutes.
9. Gently remove the adhesive film from the plate once the incubation period is complete and before placing the plate into the luminometer.
10. Read the plate in a GloMax or equivalent luminometer.
11. Transfer the Patient and Raw data to the Turbo TSI Data Analysis Software (DAS) workbook. Results are reported as IU/L calculated based the proprietary algorithm.

### Interpretation of results

The Turbo TSI assay quantifies results in IU/L using the Thyretain Turbo TSI Data Analysis Software (DAS) workbook. The assay’s Reportable Range is from 0.021 IU/L to 11 IU/L. Samples with results less than 0.021 IU/L will be reported as “< 0.021 IU/L”. Samples with results greater than 11 IU/L will be reported as “> 11 IU/L”. The dilution of samples with results greater than 11 IU/L is not supported.

### Serum samples

Normal serum samples were obtained from Interstate Blood Bank, Inc. (Memphis, TN, USA) or from the serum bank at the Molecular Thyroid Research Laboratory, Johannes Gutenberg University (JGU) Medical Center, Mainz, Germany subsequent to written informed consent and approval of the Ethical Committee of the Rhineland-Palatinate Medical Association, number 837.214.10 (7223). All normal serum samples were tested for TSH, free T4, and free T3 and for autoantibodies to thyroperoxidase, thyroglobulin, and TSH-R. All normal serum samples were shown to be from euthyroid patients and were negative for autoantibodies to thyroid antigens. Serum samples from patients with autoimmune thyroid disease (AITD) and TSI-positive serum samples were obtained from the serum bank at the JGU Thyroid Lab.

### limits of blank, detection, and quantitation

The limits of blank (LoB), detection (LoD), and quantitation (LoQ) were determined according to the Clinical and Laboratory Standards Institute (CLSI)-approved guidelines (17, 18). LoB and LoD were calculated according to the following formulas: LoB = Mean _blank_ + 1.64 x SD _blank_ and LoD = LoB + 1.64 × mean SD _low concentration sample_. The Turbo TSI LOD was verified using WHO IS spiked into normal serum at concentrations of 0.010, 0.012, 0.016 and to 0.018 IU/L. Twenty-six samples at each concentration were tested. The LoQ was determined using World Health Organization (WHO) International Standard (IS) spiked samples at concentrations between 0.02 and 0.03 IU/L. Five spiked samples were tested in 12 replicates each, across two lots of Turbo TSI bioassay over three days. The LoQ was determined from the lowest sample concentration meeting accuracy criteria (%TE ≤ 45%) where Total Error = Bias + 1.96 x Precision (%CV).

### Turbo TSI cut-off

The Turbo TSI cut-off was determined using 198 serum samples that were classified as positive or negative for TSI by the Thyretain**^®^** TSI bioassay.

### Cross reactivity

Twenty-one blood components, hormones and therapeutic drugs were tested at supraphysiologic concentrations. Serum obtained from patients with 12 disease states were tested (182 total samples).

### Interference testing

The same 21 blood components, hormones and therapeutic drugs that were tested for cross reactivity were tested for interference of the Turbo TSI assay. Each substance was mixed with a TSI-positive serum (0.233 IU/L) sample and tested in the Turbo TSI assay. The percent difference between the measurement with and without the substance was determined. Fifty-eight serum samples from patients with various disease states were also tested for Interference in Turbo TSI assay by mixing them with a low TSI positive (0.178 IU/L) serum.

### Linearity

The Turbo TSI assay was run at 17 concentrations from 0.19 to 12.00 IU/L using 5 replicates per concentration.

### Assay precision

Five concentrations of positive TSI samples from 0.047 to 9.807 IU/L were tested by 2 operators using two replicates over 20 days (n=80).

### Reproducibility

A panel of TSI-positive serum samples were tested in the Turbo TSI bioassay by two operators at three locations over 5 days.

### Sample and kit stability

TSI-positive samples at five concentrations were stored at the indicated temperatures for the indicated periods of time days before testing. Three different concentrations of TSI serum samples were tested by three lots of Turbo TSI kit once a month up to 16 months. The stability of the TSI kit was evaluated using the recommended approach in the CLSI-EP25 guidelines. A scatter plot containing a linear regression line was generated to visualize the calculated TSI concentration (IU/L) for each sample (y-axis) plotted against time over 16 months (x-axis).

### Comparison of Thyretain and Turbo Bioassays

Two-hundred and ninety-five serum samples from patients with AITD were tested with the Thyretain**^®^** TSI Bioassay and Turbo TSI bioassay at two sites.

## Results

### Determination of the limits of blank, detection and quantitation and assay cut-off

**Figure 1:**
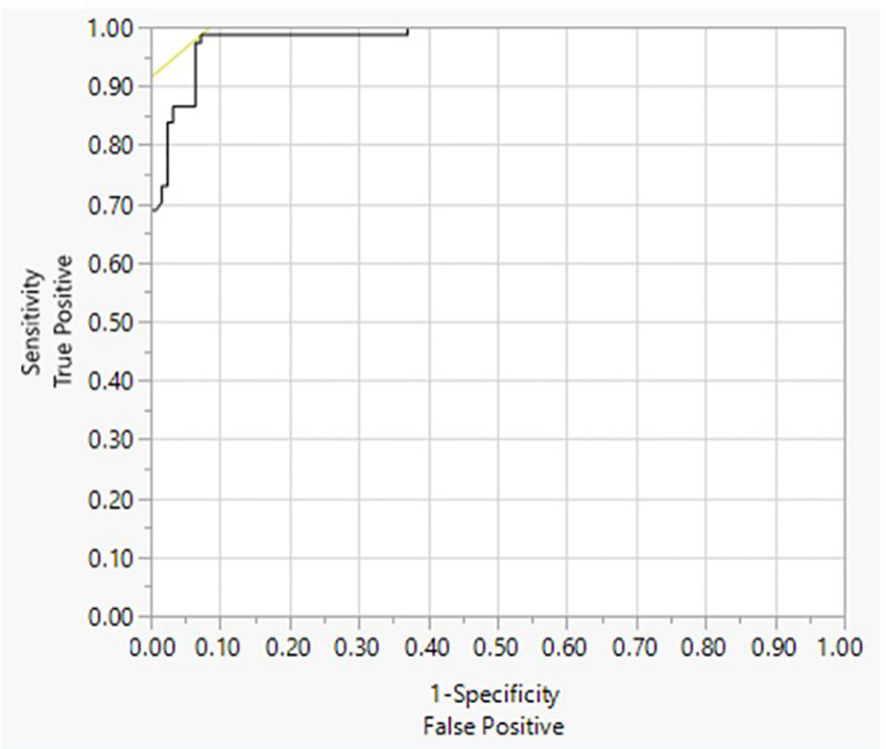
Receiver operating characteristics (ROC) of Turbo TSI. ROC analysis of the Turbo TSI bioassay. Diagnosis = 1 to be positive. Area under the curve (AUC) = 0.98363.

The LOB was found to be 0.007 IU/L. The LOD was determined to be 0.016 IU/L (**Table 1**). The LoQ was determined to be 0.021 IU/L (**Supplementary Table 1**). The Turbo TSI bioassay cutoff was determined to be 0.0241 IU/L using a ROC curve analysis, which yielded the highest true positive rate (98.7% sensitivity) together with the lowest false positive rate (93.5% specificity) against results from the Thyretain**^®^** TSI bioassay (**Figure 1**).

**Table 1:** TURBO TSI LOB^1^ and LOD^1^.

	Normal Serum spiked with WHO IS			
	0.010 IU/L	0.012 IU/L	0.016 IU/L	0.018 IU/L
n	26	26	26	26
Measured TSI Conc	0.005 IU/L	0.009 IU/L	0.015 IU/L	0.016 IU/L
St Dev	0.003	0.004	0.004	0.004
%CV	68%	43%	29%	25%
% Above LoB	23%	69%	100%	100%

^1^Limit of blank and limit of detection. Turbo TSI LoB = 0.007 IU/L. LoD_tentative_ = LoB+1.647xSD_low positive_ = 0.014 IU/L. Verified LoD = 0.016 IU/L. n = number St Dev = standard deviation %CV = percent coefficient of variation

### Cross reactivity and interference testing

The TSI concentrations measured for each of the tested blood components, hormones and drugs were all below the Turbo TSI assay cutoff (0.0241 IU/L), indicating there was no cross-reactivity (**Table 2**). Of the 182 serum samples from patients with various disease states and autoantibodies all but seven were below the Turbo TSI cut-off of 0.0241 IU/L (1/22 with type 1 diabetes; 2/50 celiac disease, and 4/21 thyroid cancer) (**Supplementary Table 2**). Six of these seven positive samples were positive in the Thyretain**^®^** TSI bioassay (data not shown). The same blood components, hormones and drugs were tested in interference studies and the percent change for each tested substance was ≤15% (**Supplementary Table 3**). None of the sera from patients with various disease states or autoantibodies interfered with the Turbo TSI assay (**Supplementary Table 4**).

**Table 2:** TURBO TSI: Cross-reactivity of blood components and drugs.

Cross Reactant	Concentration	Turbo TSI (IU/L)	< Cut-off?
Hemoglobin	1000 mg/dL	0.000	Yes
Bilirubin	40 mg/dL	0.000	Yes
Luteinizing hormone (LH)	500 IU/L	0.005	Yes
Thyroid-stimulating hormone (TSH)	100 mIU/L	0.000	Yes
Follicle-stimulating hormone (FSH)	750 IU/L	0.000	Yes
Human chorionic gonadotropin (hCG)	100,000 IU/L	0.000	Yes
Estradiol	4.5 ng/mL	0.000	Yes
Progesterone	2.2 µug/mL	0.000	Yes
Prolactin	3.9 ug/mL	0.002	Yes
Levothyroxine	1.6 ug/mL	0.003	Yes
Liothyronine	20 ug/mL	0.000	Yes
Selenium	2 ug/mL	0.000	Yes
6-propyl-2-thiouracil	91 ug/mL	0.000	Yes
Lithium	75 ug/mL	0.000	Yes
Interferon α 2B	4.4 ng/mL	0.000	Yes
Interleukin-2 human	14 ug/mL	0.001	Yes
Iodine	1 ug/mL	0.000	Yes
Sunitinib malate	200 ug/mL	0.000	Yes
Imatinib mesylate	26 ug/mL	0.000	Yes
Ipilimumab	580 µg/mL	0.006	Yes
Alemtuzumab	264 µg/mL	0.006	Yes

### Linearity and precision

The Turbo TSI assay demonstrated linearity from 0.015 to 11.958 IU/L, as all samples showed ±10% deviation from the expected linear relationship (**Supplementary Table 5**). The overall within-laboratory precision was ≤15%CV for all samples (**Table 3**).

**Table 3:** TURBO TSI: Precision^1^.

Mean Concentration (IU/L)	N	Repeatability		Between Run		Between Day		Overall Precision	
		SD	%CV	SD	%CV	SD	%CV	SD	%CV
0.047	80	0.003	6.74%	0.002	4.17%	0.003	6.60%	0.005	10.32%
0.255	80	0.006	2.39%	0.012	4.55%	0.000	0.00%	0.013	5.14%
2.837	80	0.061	2.15%	0.132	4.64%	0.000	0.00%	0.145	5.11%
5.570	80	0.144	2.58%	0.253	4.54%	0.000	0.00%	0.291	5.23%
9.807	80	0.512	5.22%	0.607	6.19%	0.146	1.49%	0.808	8.24%

^1^Serum samples at each the 5 indicated TSI concentrations were tested using 2 replicates (repeatability) and by 2 operators (between run) over 20 days (between day) (n=80). Overall precision is based on all 80 tests at each concentration. n = number St Dev = standard deviation %CV = percent coefficient of variation

### Sample and kit stability

TSI serum samples stored at different temperatures (15°C, 23°C, and 30°C) are stable for up to 4 days (**Supplementary Table 6**). TSI serum samples were stable at -70°C for up to at least 14 months. The samples also can be stored at -20°C for 3 months, or at 4°C for 3 weeks (**Supplementary Table 7**).

Overall quantification (IU/L) for all serum samples measured over 16 months using three different kit lots showed a %CV ≤14% and, based on a regression analysis, no significant measurement drift was observed under the storage conditions for all Turbo TSI kits (**Supplementary Table 8**).

### Reproducibility

The overall reproducibility of Turbo TSI assay was %CV ≤20 for five concentrations (0.06 to 5.16 IU/L) (**Supplementary Table 9**).

### Temperature tolerability

A sample panel containing five clinical TSI positive specimens were tested at different temperatures. The Turbo TSI assay performed in the temperature range between 18°C and 26°C yielded consistent results with %CV ≤20% for all controls and samples (**Supplementary Table 10**).

### Comparison with Thyretain TSI bioassay

Turbo TSI bioassay demonstrated 95.2% positive percent agreement and 94.8% negative percent agreement when compared to the Thyretain**^®^** TSI Reporter bioassay using 295 serum samples (**Table 4**). All samples were tested with a TRAb immunoassay (Kronus, Inc., Star, ID, USA) and there was 89% concordance between the TRAb assay and the two bioassays (data not shown).

**Table 4:** Comparison of Turbo TSI and Thyretain TSI Bioassays^1^.

N = 295		Thyretain TSI		
		+	–	Invalid
Turbo TSI	**+**	79	11	0
	**–**	4	201	0
	Invalid	0	0	0
		95% Confidence Interval		
PPA	95.2% (79/83)	88.3% - 98.1%		
NPA	94.8% (201/212)	(90.9% - 97.1%)		

^1^ 295 serum samples from patients with AITD PPA, positive percent agreement; NPA, negative percent agreement. n = number

## Discussion

The present report describes in detail the analytical performance and validation of a novel, rapid, and sensitive cell-based bioassay, Turbo TSI, for the measurement of TSH-R-Ab with functional stimulating activity. This new bioassay demonstrates excellent analytical sensitivity, precision, reproducibility, and linear range. Problems with interference and cross-reactivity were not observed. The assay is robust in that kits were quite stable and serum could be stored for long periods of time at various temperatures. In addition, the assay is performed at ambient temperature and can be performed at 20-25^0^ C (68-77^0^ F), thus obviating the need for an incubator. Another noteworthy feature of the Turbo TSI bioassay is that it is a quantitative assay and results are reported in IU/L.

The Turbo TSI bioassay was compared with the FDA-cleared Thyretain**^®^** TSI and exhibited excellent positive and negative percent agreement for detecting TSI. Furthermore, the clinical application of this rapid, sensitive, and user-friendly bioassay was recently demonstrated within a prospective, controlled trial of patients with AITD (19). In this comparative study of TSH-R-Ab binding and bioassays, the TSI results of the Turbo TSI assay closely correlated with the phenotype and clinical activity and severity of patients with Graves’ hyperthyroidism and extrathyroidal manifestations.

TSH-R-Ab are important biomarkers in patients with autoimmune thyroid disease (AITD), e.g. Graves’ thyroidal and extrathyroidal disease (20). These specific autoantibodies are unique in that they can affect the function of the thyroid (4, 21). Guidelines of the European (ETA) and American (ATA) Thyroid associations recommend testing for TSH-R-Ab in patients suspected of having autoimmune hyperthyroidism (3). There are a number of *in vitro* diagnostic tests available to measure TSH-R-Ab (21). Most of these are different configurations of immunoassays that measure binding of antibodies to the TSH-R. None of these assays the measures the functional activity of TRAb. Many bioassays have been described that measure the thyroid stimulatory activity of TSH-R-Ab (8). Except for one FDA-cleared product, most of these bioassays are research tools and have not been submitted to full validation studies.

Validation studies of TSI and TBI bioassays have been previously reported by our group (14, 22). Turbo TSI is based on an engineered cell line that constitutively expresses both a human TSH-R and a unique cyclic AMP-dependent luciferase (15, 23, 24). The Turbo TSI test has a number of features that offer clinical laboratories the opportunity to measure TSI without the usual complexity and long turn-around-time of bioassays. First, the cells are used immediately after thawing, and thus no cell culturing is necessary. Second, the TSI-induced cyclic AMP activates the luciferase very quickly which shortens the incubation time. Third, the luciferin substrate enters the cells and thus, in contrast to all other TSI bioassays, a lysis step is not required prior to measuring light production. These advantages, however, are moot if the assay does not exhibit the requisite performance in validation studies which the present study has shown.

In conclusion, the development and validation of an easy-to-perform, but sensitive, reproducible, and quantitative TSI bioassay, offers a significant advancement for the evaluation of patients with AITD. There are many clinical circumstances in which knowing the functional activity of TSH-R-Ab is important and actionable. In addition, because of its simple procedure and rapid results, the Turbo TSI bioassay may allow routine assessment of TSI in patients with autoimmune hyperthyroidism.

## Data Availability

The original contributions presented in the study are included in the article/**Supplementary Material**. Further inquiries can be directed to the corresponding author/s.
