# Supplementary material for: Analytical validation of a novel bioassay for thyroid-stimulating immunoglobulin
Source: Front Endocrinol (Lausanne). 2025 Jan 7;15:1468768. doi: 10.3389/fendo.2024.1468768 (PMC11746106; doi:10.3389/fendo.2024.1468768)
Supplement: Supplementary file 1 [file Presentation1.pptx]

## Slide 1
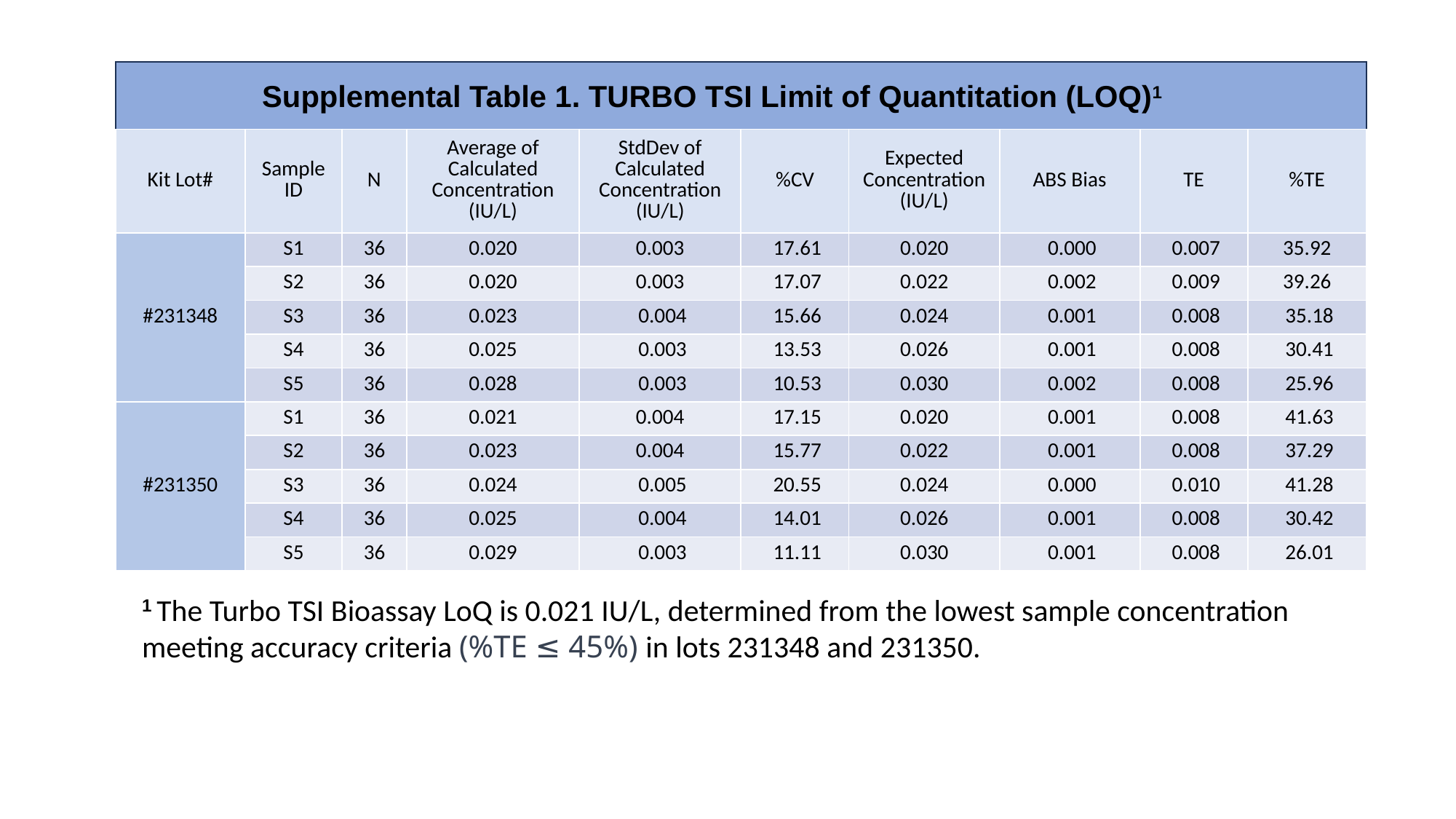

Supplemental Table 1. TURBO TSI Limit of Quantitation (LOQ)1
| Kit Lot# | Sample ID | N | Average of Calculated Concentration (IU/L) | StdDev of Calculated Concentration (IU/L) | %CV | Expected Concentration (IU/L) | ABS Bias | TE | %TE |
| --- | --- | --- | --- | --- | --- | --- | --- | --- | --- |
| #231348 | S1 | 36 | 0.020 | 0.003 | 17.61 | 0.020 | 0.000 | 0.007 | 35.92 |
| | S2 | 36 | 0.020 | 0.003 | 17.07 | 0.022 | 0.002 | 0.009 | 39.26 |
| | S3 | 36 | 0.023 | 0.004 | 15.66 | 0.024 | 0.001 | 0.008 | 35.18 |
| | S4 | 36 | 0.025 | 0.003 | 13.53 | 0.026 | 0.001 | 0.008 | 30.41 |
| | S5 | 36 | 0.028 | 0.003 | 10.53 | 0.030 | 0.002 | 0.008 | 25.96 |
| #231350 | S1 | 36 | 0.021 | 0.004 | 17.15 | 0.020 | 0.001 | 0.008 | 41.63 |
| | S2 | 36 | 0.023 | 0.004 | 15.77 | 0.022 | 0.001 | 0.008 | 37.29 |
| | S3 | 36 | 0.024 | 0.005 | 20.55 | 0.024 | 0.000 | 0.010 | 41.28 |
| | S4 | 36 | 0.025 | 0.004 | 14.01 | 0.026 | 0.001 | 0.008 | 30.42 |
| | S5 | 36 | 0.029 | 0.003 | 11.11 | 0.030 | 0.001 | 0.008 | 26.01 |
1 The Turbo TSI Bioassay LoQ is 0.021 IU/L, determined from the lowest sample concentration meeting accuracy criteria (%TE ≤ 45%) in lots 231348 and 231350.

## Slide 2
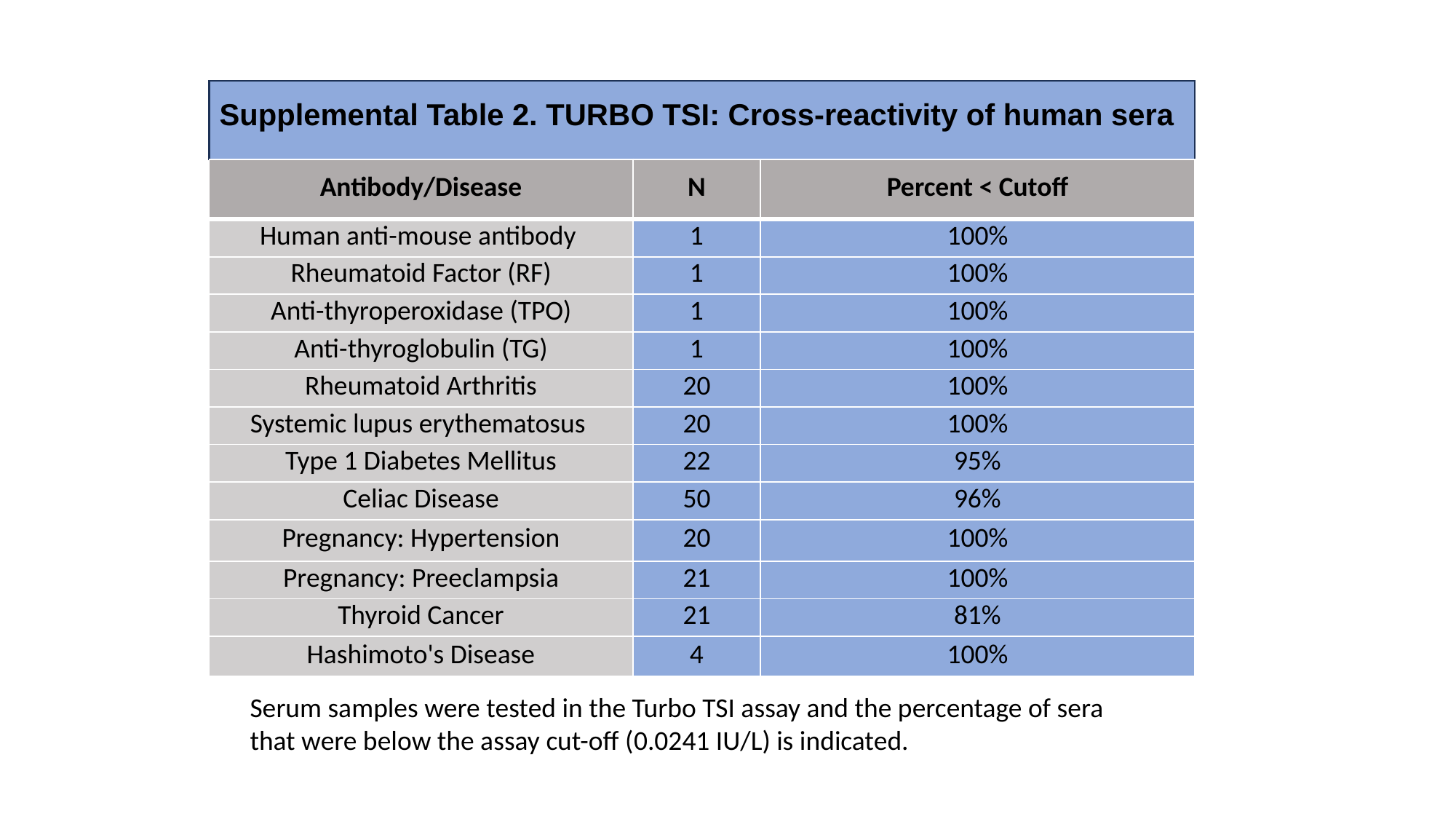

Supplemental Table 2. TURBO TSI: Cross-reactivity of human sera
| Antibody/Disease | N | Percent < Cutoff |
| --- | --- | --- |
| Human anti-mouse antibody | 1 | 100% |
| Rheumatoid Factor (RF) | 1 | 100% |
| Anti-thyroperoxidase (TPO) | 1 | 100% |
| Anti-thyroglobulin (TG) | 1 | 100% |
| Rheumatoid Arthritis | 20 | 100% |
| Systemic lupus erythematosus | 20 | 100% |
| Type 1 Diabetes Mellitus | 22 | 95% |
| Celiac Disease | 50 | 96% |
| Pregnancy: Hypertension | 20 | 100% |
| Pregnancy: Preeclampsia | 21 | 100% |
| Thyroid Cancer | 21 | 81% |
| Hashimoto's Disease | 4 | 100% |
Serum samples were tested in the Turbo TSI assay and the percentage of sera that were below the assay cut-off (0.0241 IU/L) is indicated.

## Slide 3
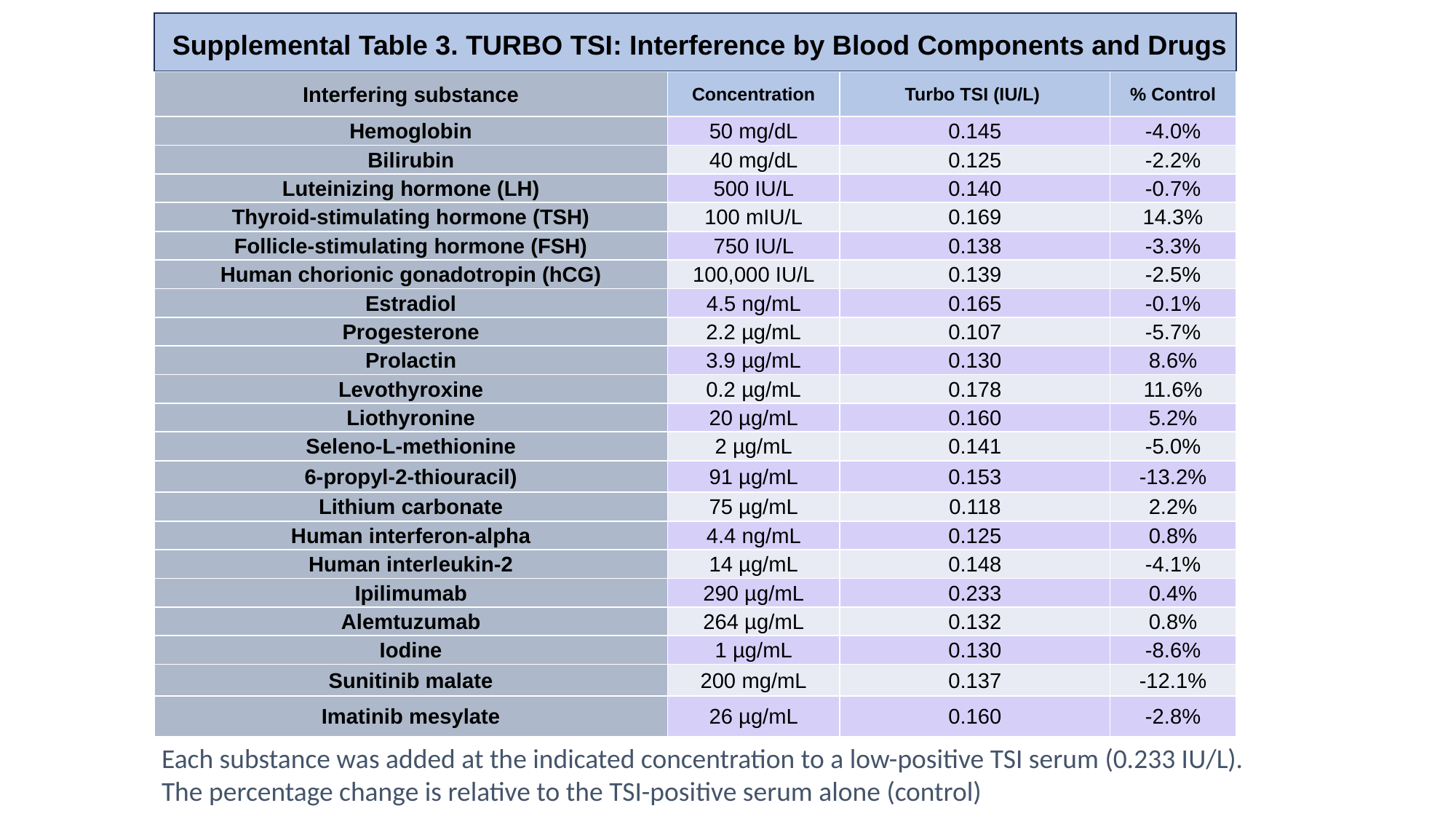

Supplemental Table 3. TURBO TSI: Interference by Blood Components and Drugs
| Interfering substance | Concentration | Turbo TSI (IU/L) | % Control |
| --- | --- | --- | --- |
| Hemoglobin | 50 mg/dL | 0.145 | -4.0% |
| Bilirubin | 40 mg/dL | 0.125 | -2.2% |
| Luteinizing hormone (LH) | 500 IU/L | 0.140 | -0.7% |
| Thyroid-stimulating hormone (TSH) | 100 mIU/L | 0.169 | 14.3% |
| Follicle-stimulating hormone (FSH) | 750 IU/L | 0.138 | -3.3% |
| Human chorionic gonadotropin (hCG) | 100,000 IU/L | 0.139 | -2.5% |
| Estradiol | 4.5 ng/mL | 0.165 | -0.1% |
| Progesterone | 2.2 µg/mL | 0.107 | -5.7% |
| Prolactin | 3.9 µg/mL | 0.130 | 8.6% |
| Levothyroxine | 0.2 µg/mL | 0.178 | 11.6% |
| Liothyronine | 20 µg/mL | 0.160 | 5.2% |
| Seleno-L-methionine | 2 µg/mL | 0.141 | -5.0% |
| 6-propyl-2-thiouracil) | 91 µg/mL | 0.153 | -13.2% |
| Lithium carbonate | 75 µg/mL | 0.118 | 2.2% |
| Human interferon-alpha | 4.4 ng/mL | 0.125 | 0.8% |
| Human interleukin-2 | 14 µg/mL | 0.148 | -4.1% |
| Ipilimumab | 290 µg/mL | 0.233 | 0.4% |
| Alemtuzumab | 264 µg/mL | 0.132 | 0.8% |
| Iodine | 1 µg/mL | 0.130 | -8.6% |
| Sunitinib malate | 200 mg/mL | 0.137 | -12.1% |
| Imatinib mesylate | 26 µg/mL | 0.160 | -2.8% |
Each substance was added at the indicated concentration to a low-positive TSI serum (0.233 IU/L). The percentage change is relative to the TSI-positive serum alone (control)

## Slide 4
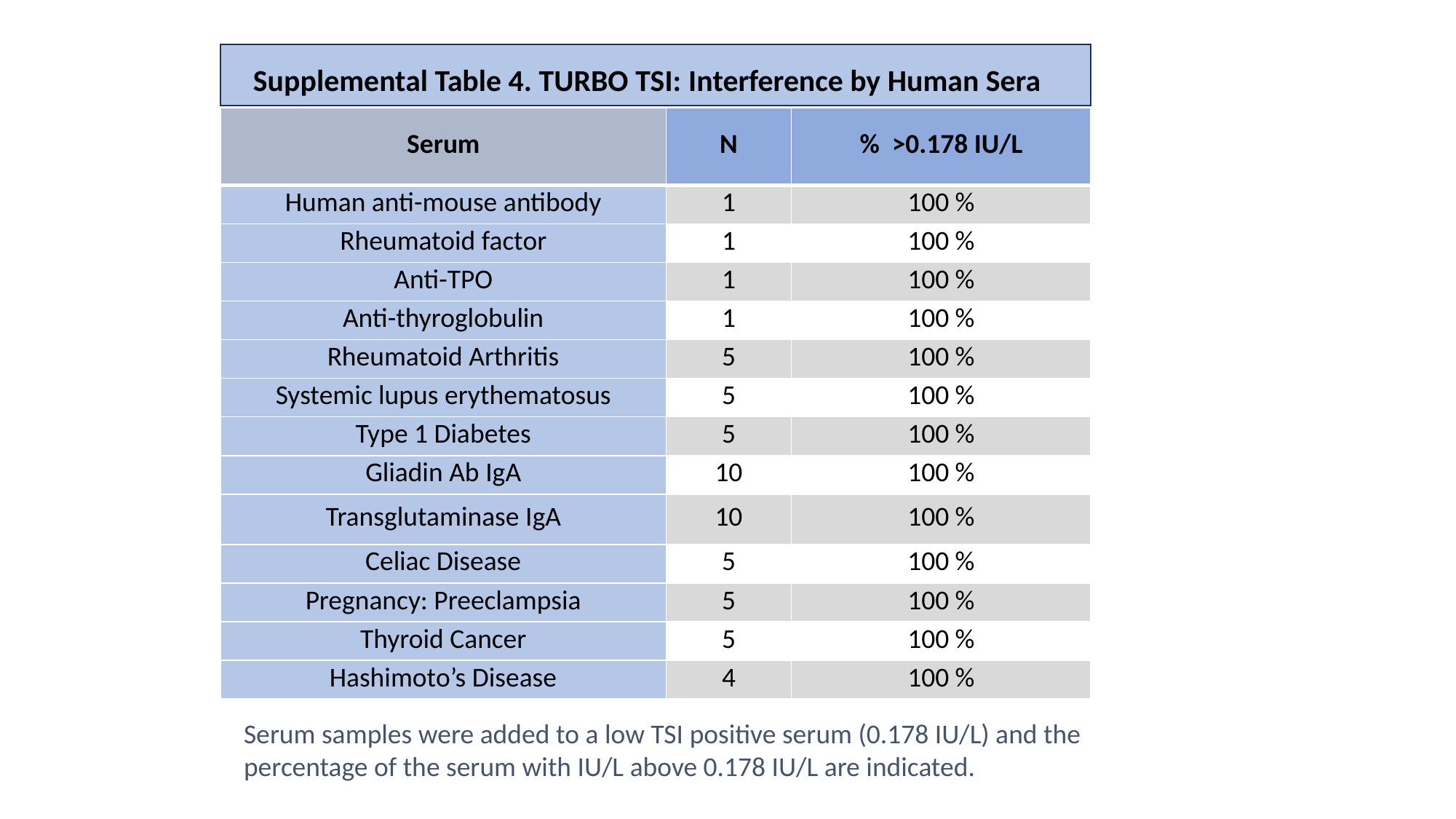

Supplemental Table 4. TURBO TSI: Interference by Human Sera
| Serum | N | % >0.178 IU/L |
| --- | --- | --- |
| Human anti-mouse antibody | 1 | 100 % |
| Rheumatoid factor | 1 | 100 % |
| Anti-TPO | 1 | 100 % |
| Anti-thyroglobulin | 1 | 100 % |
| Rheumatoid Arthritis | 5 | 100 % |
| Systemic lupus erythematosus | 5 | 100 % |
| Type 1 Diabetes | 5 | 100 % |
| Gliadin Ab IgA | 10 | 100 % |
| Transglutaminase IgA | 10 | 100 % |
| Celiac Disease | 5 | 100 % |
| Pregnancy: Preeclampsia | 5 | 100 % |
| Thyroid Cancer | 5 | 100 % |
| Hashimoto’s Disease | 4 | 100 % |
Serum samples were added to a low TSI positive serum (0.178 IU/L) and the percentage of the serum with IU/L above 0.178 IU/L are indicated.

## Slide 5
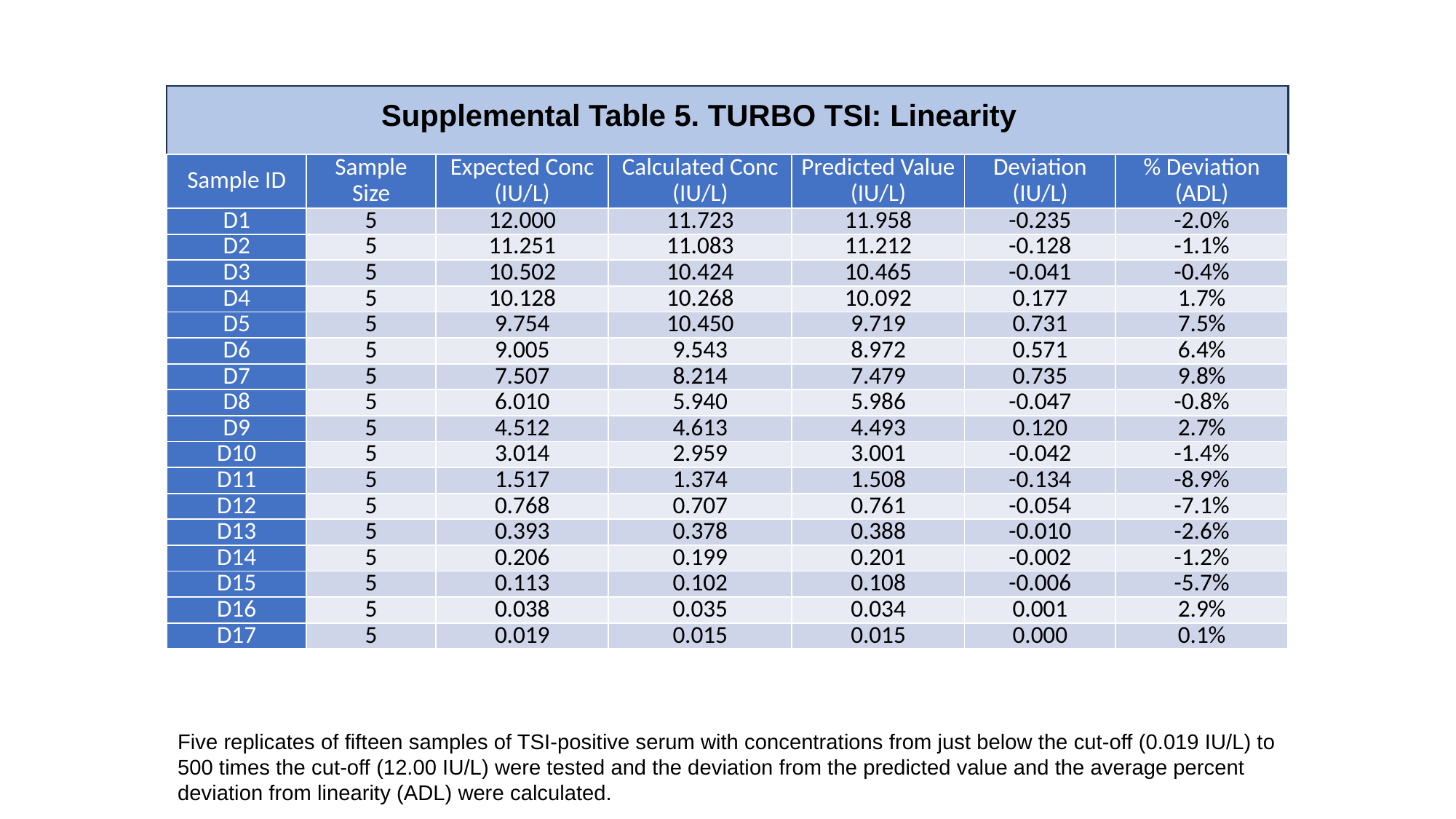

Supplemental Table 5. TURBO TSI: Linearity
| Sample ID | Sample Size | Expected Conc (IU/L) | Calculated Conc (IU/L) | Predicted Value (IU/L) | Deviation (IU/L) | % Deviation (ADL) |
| --- | --- | --- | --- | --- | --- | --- |
| D1 | 5 | 12.000 | 11.723 | 11.958 | -0.235 | -2.0% |
| D2 | 5 | 11.251 | 11.083 | 11.212 | -0.128 | -1.1% |
| D3 | 5 | 10.502 | 10.424 | 10.465 | -0.041 | -0.4% |
| D4 | 5 | 10.128 | 10.268 | 10.092 | 0.177 | 1.7% |
| D5 | 5 | 9.754 | 10.450 | 9.719 | 0.731 | 7.5% |
| D6 | 5 | 9.005 | 9.543 | 8.972 | 0.571 | 6.4% |
| D7 | 5 | 7.507 | 8.214 | 7.479 | 0.735 | 9.8% |
| D8 | 5 | 6.010 | 5.940 | 5.986 | -0.047 | -0.8% |
| D9 | 5 | 4.512 | 4.613 | 4.493 | 0.120 | 2.7% |
| D10 | 5 | 3.014 | 2.959 | 3.001 | -0.042 | -1.4% |
| D11 | 5 | 1.517 | 1.374 | 1.508 | -0.134 | -8.9% |
| D12 | 5 | 0.768 | 0.707 | 0.761 | -0.054 | -7.1% |
| D13 | 5 | 0.393 | 0.378 | 0.388 | -0.010 | -2.6% |
| D14 | 5 | 0.206 | 0.199 | 0.201 | -0.002 | -1.2% |
| D15 | 5 | 0.113 | 0.102 | 0.108 | -0.006 | -5.7% |
| D16 | 5 | 0.038 | 0.035 | 0.034 | 0.001 | 2.9% |
| D17 | 5 | 0.019 | 0.015 | 0.015 | 0.000 | 0.1% |
Five replicates of fifteen samples of TSI-positive serum with concentrations from just below the cut-off (0.019 IU/L) to 500 times the cut-off (12.00 IU/L) were tested and the deviation from the predicted value and the average percent deviation from linearity (ADL) were calculated.

## Slide 6
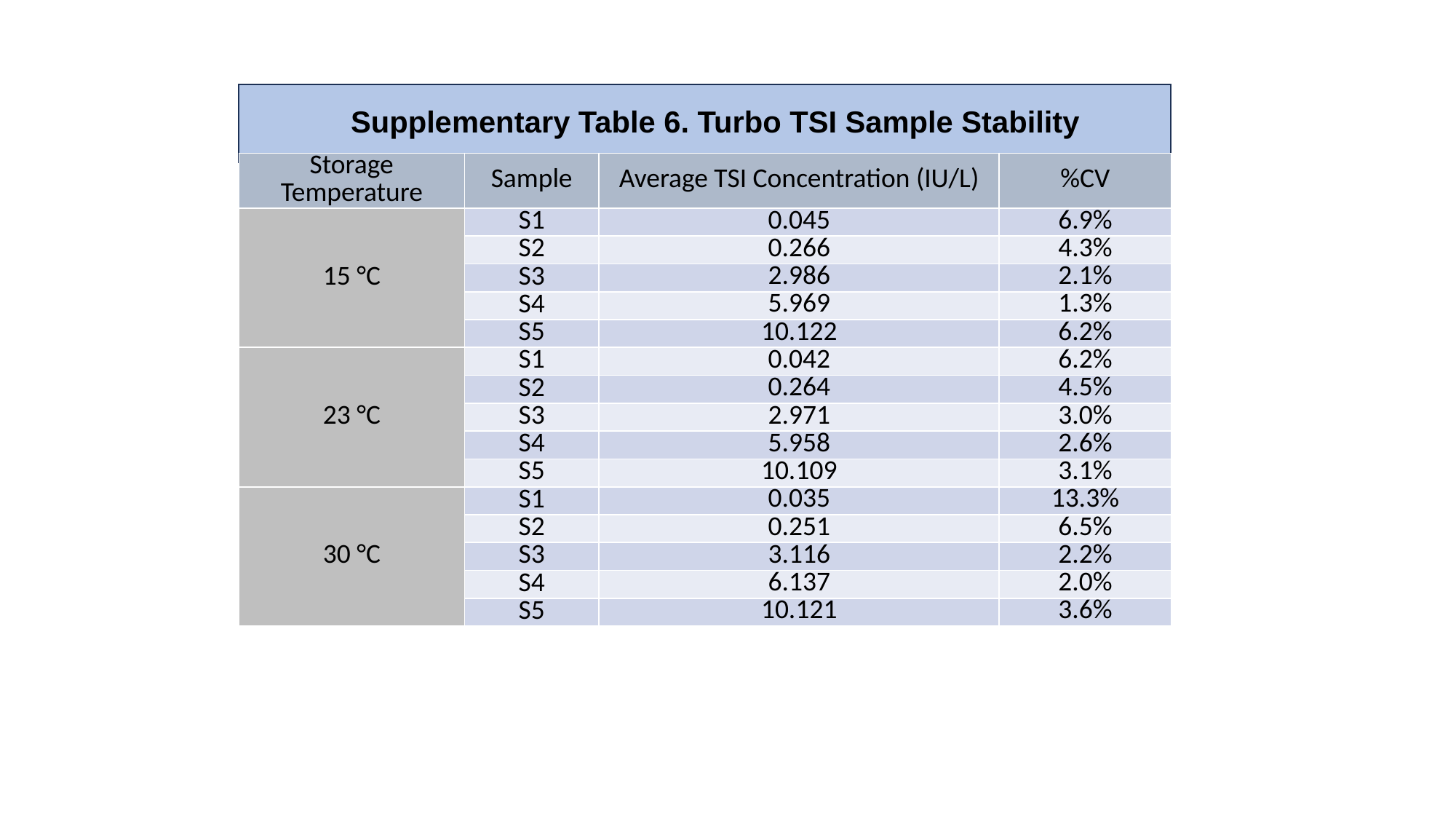

Supplementary Table 6. Turbo TSI Sample Stability
| Storage Temperature | Sample | Average TSI Concentration (IU/L) | %CV |
| --- | --- | --- | --- |
| 15 °C | S1 | 0.045 | 6.9% |
| | S2 | 0.266 | 4.3% |
| | S3 | 2.986 | 2.1% |
| | S4 | 5.969 | 1.3% |
| | S5 | 10.122 | 6.2% |
| 23 °C | S1 | 0.042 | 6.2% |
| | S2 | 0.264 | 4.5% |
| | S3 | 2.971 | 3.0% |
| | S4 | 5.958 | 2.6% |
| | S5 | 10.109 | 3.1% |
| 30 °C | S1 | 0.035 | 13.3% |
| | S2 | 0.251 | 6.5% |
| | S3 | 3.116 | 2.2% |
| | S4 | 6.137 | 2.0% |
| | S5 | 10.121 | 3.6% |

## Slide 7
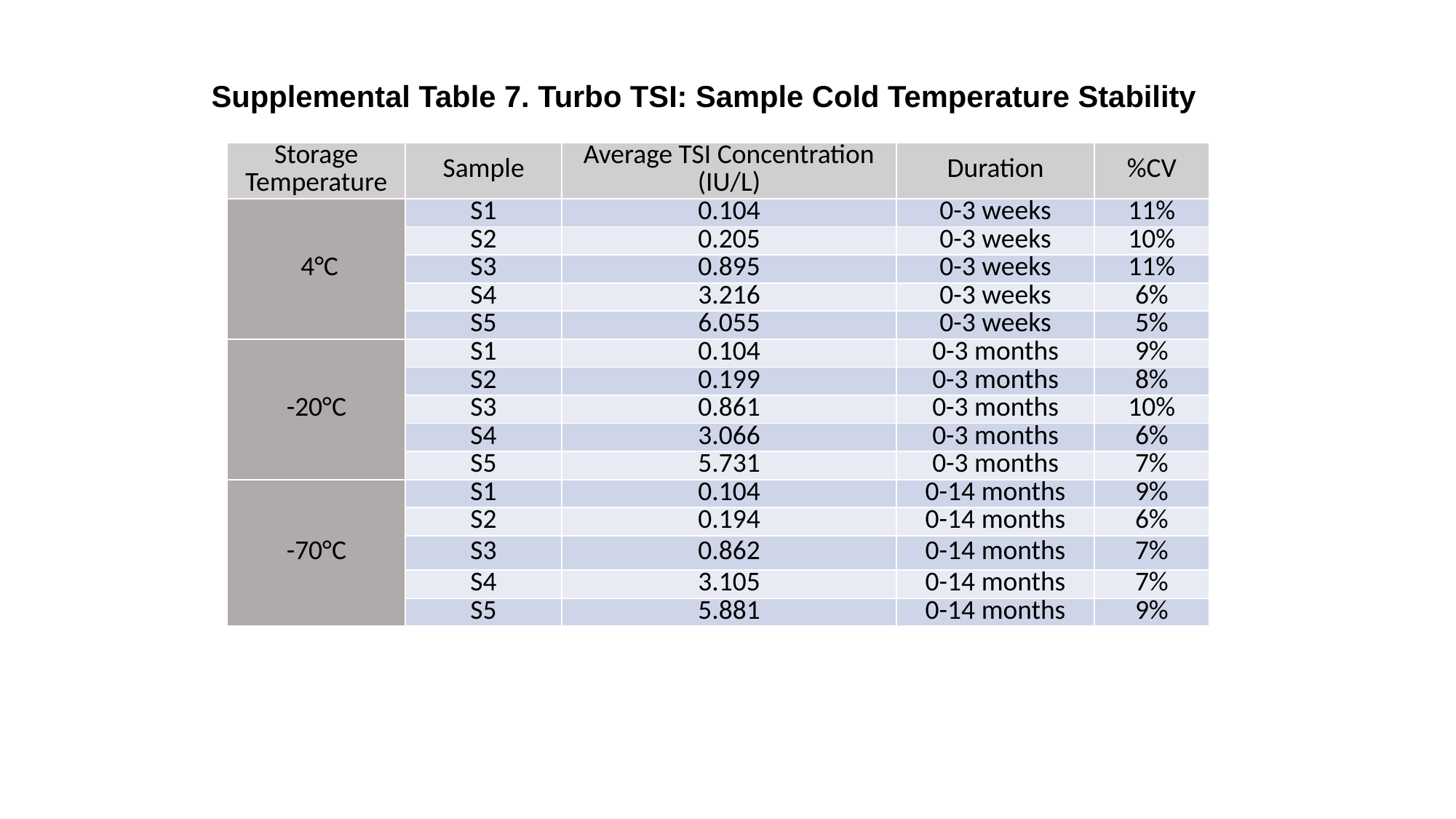

Supplemental Table 7. Turbo TSI: Sample Cold Temperature Stability
| Storage Temperature | Sample | Average TSI Concentration (IU/L) | Duration | %CV |
| --- | --- | --- | --- | --- |
| 4°C | S1 | 0.104 | 0-3 weeks | 11% |
| | S2 | 0.205 | 0-3 weeks | 10% |
| | S3 | 0.895 | 0-3 weeks | 11% |
| | S4 | 3.216 | 0-3 weeks | 6% |
| | S5 | 6.055 | 0-3 weeks | 5% |
| -20°C | S1 | 0.104 | 0-3 months | 9% |
| | S2 | 0.199 | 0-3 months | 8% |
| | S3 | 0.861 | 0-3 months | 10% |
| | S4 | 3.066 | 0-3 months | 6% |
| | S5 | 5.731 | 0-3 months | 7% |
| -70°C | S1 | 0.104 | 0-14 months | 9% |
| -70°C | S2 | 0.194 | 0-14 months | 6% |
| | S3 | 0.862 | 0-14 months | 7% |
| | S4 | 3.105 | 0-14 months | 7% |
| | S5 | 5.881 | 0-14 months | 9% |

## Slide 8
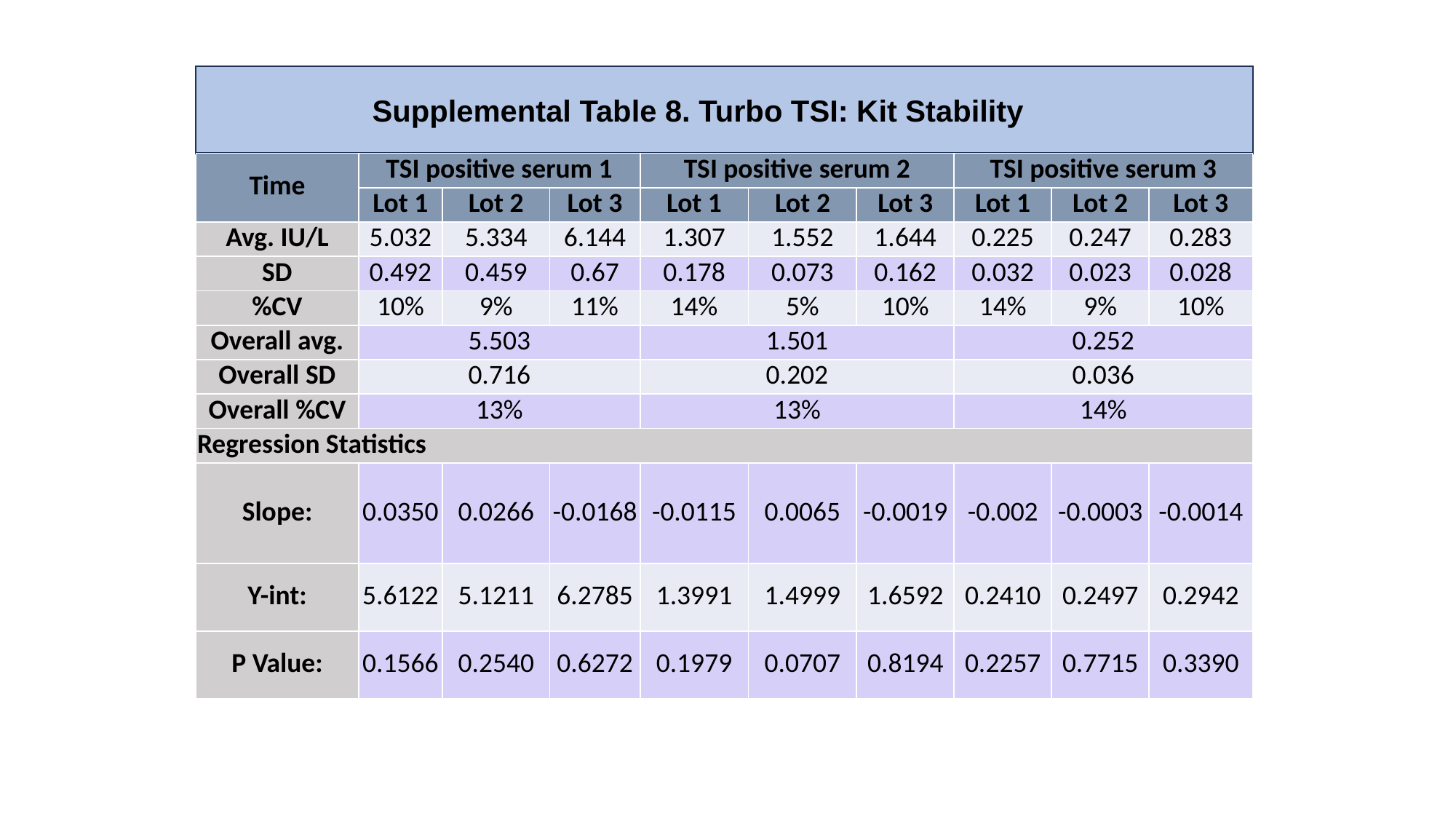

Supplemental Table 8. Turbo TSI: Kit Stability
| Time | TSI positive serum 1 | | | TSI positive serum 2 | | | TSI positive serum 3 | | |
| --- | --- | --- | --- | --- | --- | --- | --- | --- | --- |
| | Lot 1 | Lot 2 | Lot 3 | Lot 1 | Lot 2 | Lot 3 | Lot 1 | Lot 2 | Lot 3 |
| Avg. IU/L | 5.032 | 5.334 | 6.144 | 1.307 | 1.552 | 1.644 | 0.225 | 0.247 | 0.283 |
| SD | 0.492 | 0.459 | 0.67 | 0.178 | 0.073 | 0.162 | 0.032 | 0.023 | 0.028 |
| %CV | 10% | 9% | 11% | 14% | 5% | 10% | 14% | 9% | 10% |
| Overall avg. | 5.503 | | | 1.501 | | | 0.252 | | |
| Overall SD | 0.716 | | | 0.202 | | | 0.036 | | |
| Overall %CV | 13% | | | 13% | | | 14% | | |
| Regression Statistics | | | | | | | | | |
| Slope: | 0.0350 | 0.0266 | -0.0168 | -0.0115 | 0.0065 | -0.0019 | -0.002 | -0.0003 | -0.0014 |
| Y-int: | 5.6122 | 5.1211 | 6.2785 | 1.3991 | 1.4999 | 1.6592 | 0.2410 | 0.2497 | 0.2942 |
| P Value: | 0.1566 | 0.2540 | 0.6272 | 0.1979 | 0.0707 | 0.8194 | 0.2257 | 0.7715 | 0.3390 |

## Slide 9
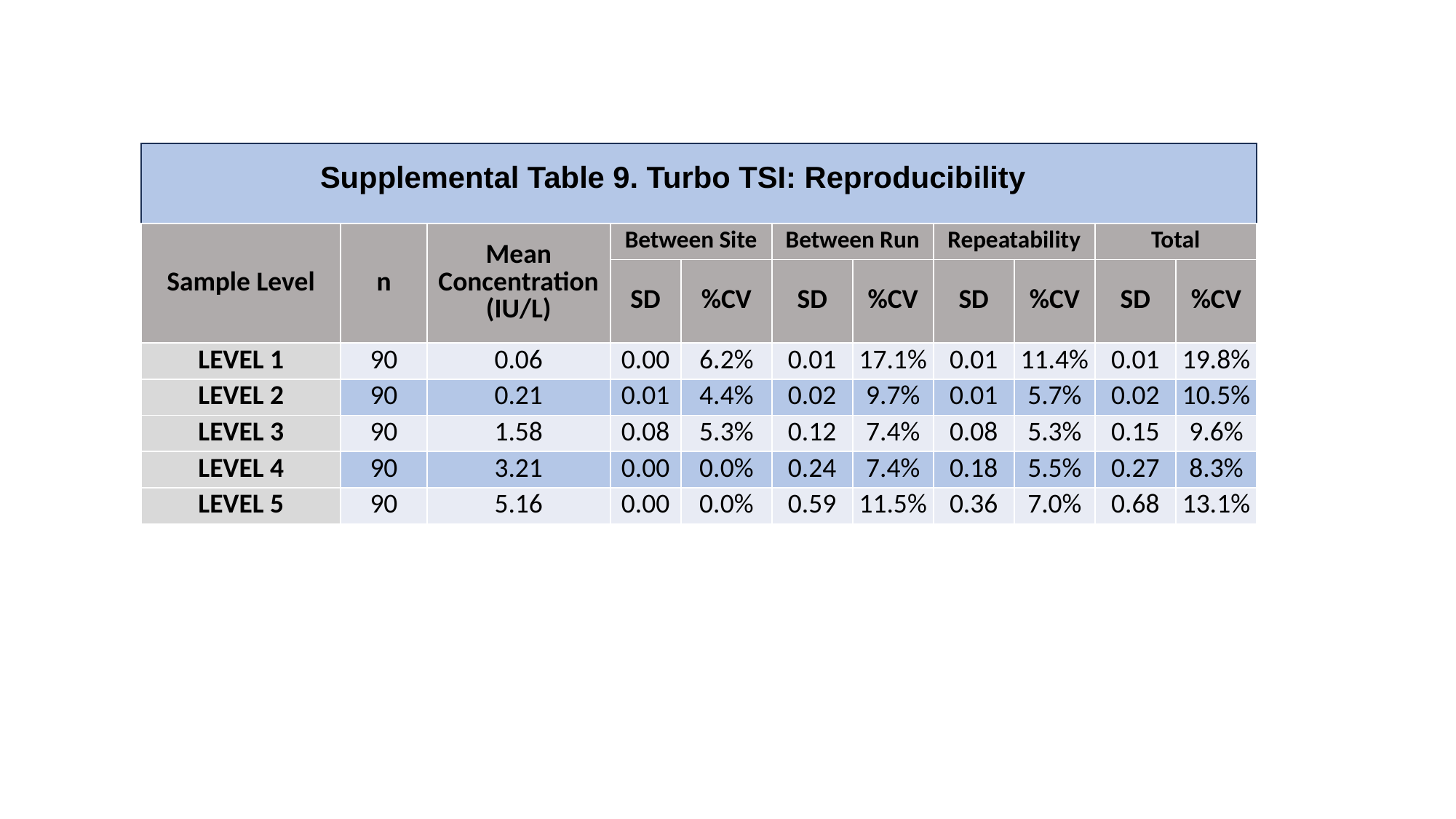

Supplemental Table 9. Turbo TSI: Reproducibility
| Sample Level | n | Mean Concentration (IU/L) | Between Site | | Between Run | | Repeatability | | Total | |
| --- | --- | --- | --- | --- | --- | --- | --- | --- | --- | --- |
| | | | SD | %CV | SD | %CV | SD | %CV | SD | %CV |
| LEVEL 1 | 90 | 0.06 | 0.00 | 6.2% | 0.01 | 17.1% | 0.01 | 11.4% | 0.01 | 19.8% |
| LEVEL 2 | 90 | 0.21 | 0.01 | 4.4% | 0.02 | 9.7% | 0.01 | 5.7% | 0.02 | 10.5% |
| LEVEL 3 | 90 | 1.58 | 0.08 | 5.3% | 0.12 | 7.4% | 0.08 | 5.3% | 0.15 | 9.6% |
| LEVEL 4 | 90 | 3.21 | 0.00 | 0.0% | 0.24 | 7.4% | 0.18 | 5.5% | 0.27 | 8.3% |
| LEVEL 5 | 90 | 5.16 | 0.00 | 0.0% | 0.59 | 11.5% | 0.36 | 7.0% | 0.68 | 13.1% |

## Slide 10
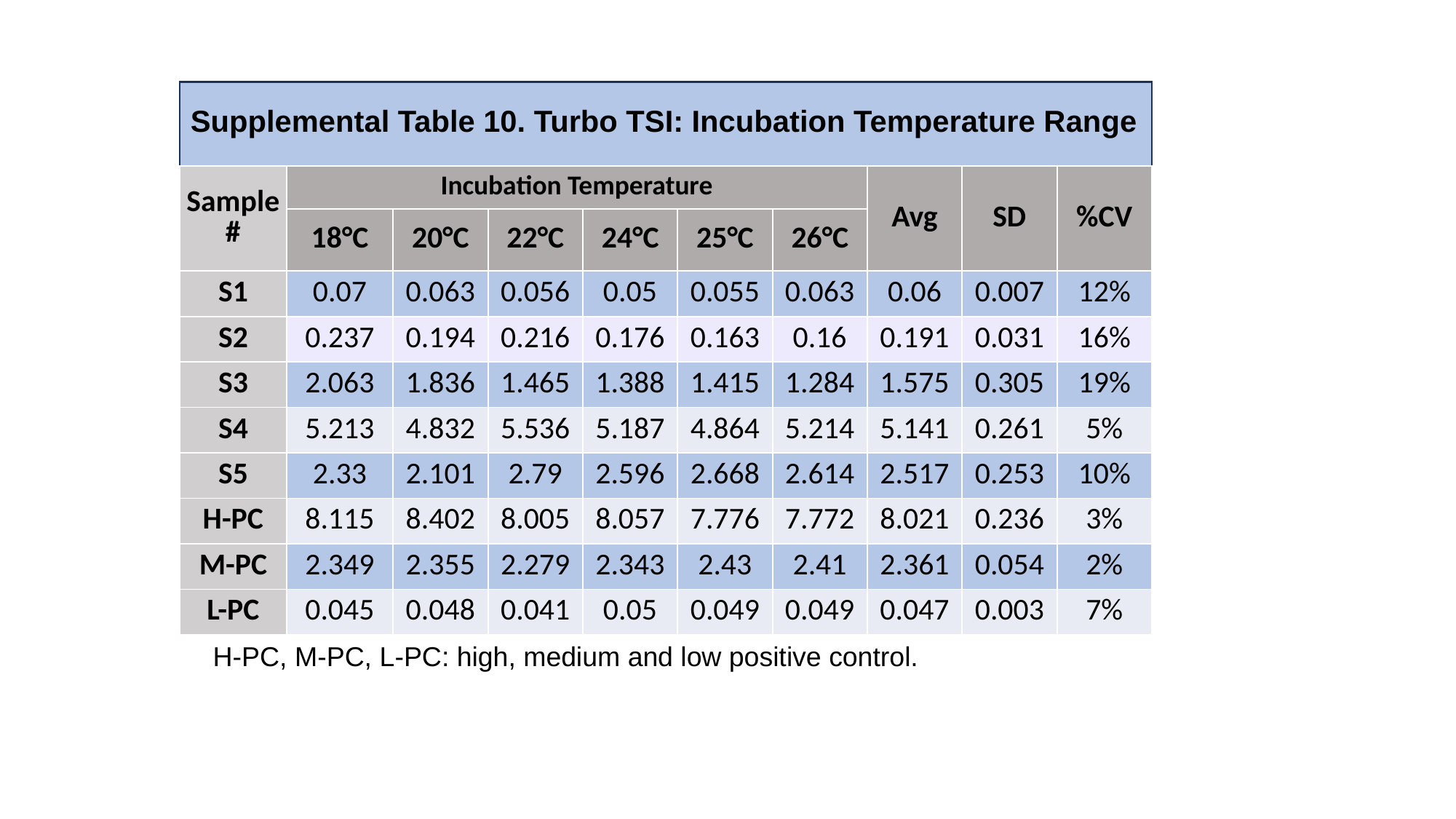

Supplemental Table 10. Turbo TSI: Incubation Temperature Range
| Sample # | Incubation Temperature | | | | | | Avg | SD | %CV |
| --- | --- | --- | --- | --- | --- | --- | --- | --- | --- |
| | 18°C | 20°C | 22°C | 24°C | 25°C | 26°C | | | |
| S1 | 0.07 | 0.063 | 0.056 | 0.05 | 0.055 | 0.063 | 0.06 | 0.007 | 12% |
| S2 | 0.237 | 0.194 | 0.216 | 0.176 | 0.163 | 0.16 | 0.191 | 0.031 | 16% |
| S3 | 2.063 | 1.836 | 1.465 | 1.388 | 1.415 | 1.284 | 1.575 | 0.305 | 19% |
| S4 | 5.213 | 4.832 | 5.536 | 5.187 | 4.864 | 5.214 | 5.141 | 0.261 | 5% |
| S5 | 2.33 | 2.101 | 2.79 | 2.596 | 2.668 | 2.614 | 2.517 | 0.253 | 10% |
| H-PC | 8.115 | 8.402 | 8.005 | 8.057 | 7.776 | 7.772 | 8.021 | 0.236 | 3% |
| M-PC | 2.349 | 2.355 | 2.279 | 2.343 | 2.43 | 2.41 | 2.361 | 0.054 | 2% |
| L-PC | 0.045 | 0.048 | 0.041 | 0.05 | 0.049 | 0.049 | 0.047 | 0.003 | 7% |
H-PC, M-PC, L-PC: high, medium and low positive control.
